# Psychiatric symptoms and emotional impact of the COVID-19 pandemic on Italian adolescents during the third lockdown: a cross-sectional cohort study

**DOI:** 10.1038/s41598-022-25358-0

**Published:** 2022-12-03

**Authors:** Martina Maria Mensi, Marta Iacopelli, Marika Orlandi, Luca Capone, Chiara Rogantini, Arianna Vecchio, Erica Casini, Renato Borgatti

**Affiliations:** 1 Child Neurology and Psychiatry Unit, IRCCS Mondino Foundation, via Mondino 2, 27100 Pavia, Italy; 2 Department of Brain and Behavioral Sciences, University of Pavia, 27100 Pavia, Italy; 3 Università degli Studi di Milano-Bicocca, 20126 Milan, Italy

**Keywords:** Psychology, Risk factors, Signs and symptoms

## Abstract

A previous study showed that about 80% of Italian adolescents reported isolated symptoms of acute or post-traumatic stress during the first lockdown in Italy. We proposed a new questionnaire to investigate the presence of symptoms related to anxiety, panic, depression, eating disorders, sleep disorders, social withdrawal, stress disorders, psychotic symptoms, anti-conservative thoughts, and self-harming acts aggravated by COVID-19 restrictions and possible differences between males and females. 500 adolescents (12–18 years) completed an online survey created using validated scales. 41% of the respondents felt more stress than during the first lockdown. 1.85% showed suprathreshold symptoms for post-traumatic stress disorder and 21% showed isolated symptoms of acute or post-traumatic stress due to the pandemic. In addition, we found psychotic symptoms (16%), panic (25% suprathreshold), anxiety (46.8% suprathreshold), depression (18.7% suprathreshold), eating-related symptoms (51%), sleep difficulties (57%), a tendency to social withdrawal after the pandemic (15%), suicidal ideation (30%), and self-harming behavior (9%). Furthermore, girls showed a more severe level of distress. The results show a high prevalence of symptoms because of the COVID-19 pandemic and confirm the need for easy access to support and treatment service to help contain the bio-psycho-social risk factors prompted by the current pandemic and promptly and effectively manage the consequences.

## Introduction

The COVID-19 pandemic is having a significant impact on the mental health of young people around the world, with heightened isolation and psychosocial distress. In the general population, depressive symptoms increased the most during the pandemic, followed by anxiety and stress-related disorders^1,2^. Many studies described depression, fatigue, low mood, irritability, insomnia, post-traumatic stress symptoms, anger, and emotional exhaustion as psychological symptoms related to the pandemic. Stressors during lockdown include the duration of quarantine, fears of infection, frustration, boredom, and inadequate supplies and information^1^. The incidence of such symptoms increased significantly in coincidence with the lockdown phases, evidence that the pandemic has led to a condition of instability and unpredictability^3^. Moreover, studies found that the pandemic affected the female population more than the male one^4–6^.

In addition to organic impairment, the COVID-19 pandemic has generated an overall change in the whole population and especially in adolescents. Recent literature has highlighted significant emotional difficulties experienced by adolescents and reactive to bio-psycho-social factors due to the ongoing pandemic^3,7–9^. Moreover, social isolation, school closures, lack of interactions, fear of infection, and feelings of loneliness have compromised the mental health of adolescents leading to an increase in the broad sense of emotional-behavioral problems and worsening psychopathological aspects^10^. In a previous study regarding the adolescent population, stress related to the COVID-19 pandemic was found to be significantly related to worse adjustment, with the presence of depression and loneliness. However, time spent with family, virtual contact with friends, and increased physical activity were found to be correlated with less loneliness and could be coping strategies aimed at reducing feelings of loneliness during social isolation^11,12^.

Other studies have brought to light the existence of a relationship between COVID-19 fear, death obsession and levels of loneliness. In particular, researchers have shown that as loneliness levels increase, death obsession also increases^13^.

There are, however, mixed results about young people's perceived fear of infection. For example, one study showed that Italian adolescents have a low perception of the risk associated with contracting COVID-19. Young people believe that it is not a potentially serious disease, and they underestimate the likelihood of contracting it, showing high confidence in their good health^14^. Additionally, anxiety, depression, irritability, boredom, inattention and fear of COVID-19 are predominant new-onset psychological problems in children^3,9,15–17^.

Other problems exacerbated by the pandemic include increased stress levels, social withdrawal, eating disorders, suicidal ideation and self-harm^18,19^. The rules of social distancing also resulted in a tendency among adolescents to become ‘invisible to society’, aspiring to ‘social death’ and ‘avoiding physical death’, preponderant characteristics in Hikikomori^20^. Some studies have also shown how the use of technological tools and social media during lockdown is a strategy for coping with restrictions imposed to limit the spread of contagion. Maintaining social connections online would alleviate adolescents' feelings of distress and anxiety to some extent by increasing feelings of happiness^21^ by satisfying the need for sociability and connection^22^.

However, other studies point out how the use of technology can significantly turn into a habit that is difficult to break^23^. In fact, other studies reveal a strong relationship between compulsive online behaviors, symptoms of depression and loneliness in adolescents^24^.

Limited access to care networks, increased exposure to the body image ideal, and lack of social relationships represent three pathways of emotional dysfunction and exacerbation of eating disorders^25–27^. Orgilés et al.^7^ tried to examine the emotional impact of quarantine on children and adolescents in Italy and Spain, showing that people suffering from mental fragility experience more distress. Moreover, Uccella et al.^9^ also demonstrated a strong relationship between the level of severity of children's dysfunctional behavior and the degree of their parents’ circumstantial distress. A previous survey confirmed that patients with pre-existing neuropsychiatric disorders show more severe stress symptoms than the general population, and worse overall functioning^28^. The same study also revealed a worrying incidence of symptoms related to acute stress disorder and post-traumatic stress disorder in Italian adolescents during the first lockdown (March 2020–May 2020), reaching 80% of subthreshold symptoms even in the healthy adolescent population. The persistence of the COVID-19 emergency and the uncertainty about the future are therefore unique experimental conditions to derive epidemiological data on the emotional impact of the COVID-19 on adolescent population and plan intervention strategies^29^. Furthermore, it is important to collect impressions during all pandemic to understand how adolescents’ functioning has changed, since the first lockdown, in response to the persistence of limitations on personal freedom. Then, during the third wave of pandemic in Italy (from March 2021 to June 2021) and the resulting new tightening of restrictive measures we proposed a new questionnaire. Restrictive measures included a ban on travel between Italian regions and abroad, all schools were closed, classes were held online only, non-essential stores remained closed, and the vaccination campaign was started. The questionnaire uncovered acute and post-traumatic stress symptoms, but also anxiety symptoms, depression symptoms, anticonservative ideas, self-harm actions, substance abuse, dysfunctional eating behaviors, sleep disorders and social withdrawal.

This study aimed to investigate:

1. The prevalence rate and sociodemographic correlates of COVID-19-related stress disorders, depressive, anxious, panic, and psychotic symptoms, according to DSM-5 criteria, during the third lockdown in Italy.
2. Adolescents’ self-perception of personal stress level, dysfunctional eating behaviors, sleep problems, substance abuse, perceived social withdrawal, and suicidal ideation and self-harm during the third lockdown in Italy.
3. Differences between male adolescents and female adolescents.

From this survey, we would expect a higher incidence of COVID-related PTSD symptoms than from the survey proposed during the first lockdown by Mensi and colleagues^20^.

We would also expect results in line with the literature regarding the presence of high percentages of anxiety and depressive symptoms, as well as a high incidence of suicidal ideation.

Finally, we would expect results in line with the literature regarding higher symptomatology in the female population.

## Methods

### Design

This clinical register-based cross-sectional observational cohort study was conducted according to the Strengthening the Reporting of Observational Studies in Epidemiology (STROBE) statement (supplementary material). The study received the approval of the Ethics Committee of Policlinico San Matteo in Pavia, Italy (P-20210060206), and was conducted following the Declaration of Helsinki (1964) and its later amendments. Every participant gave his/her written informed consent and was free to withdraw their participation in the study at any time. Parents of underage participants were not asked for informed consent, as the fact that adolescents could access digital platforms in which there is the possibility of conducting surveys presupposes parental authorization to carry them out. In addition, a compilation of consent from parents, with signatures, would have prevented anonymity. All data were anonymous and accessible only by personnel specifically trained according to procedures agreed with the principal investigator and after approval by the Ethics Committee. The dataset generated and analyzed during the current study is available in the Zenodo repository^30^.

### Participants

**Figure 1:**
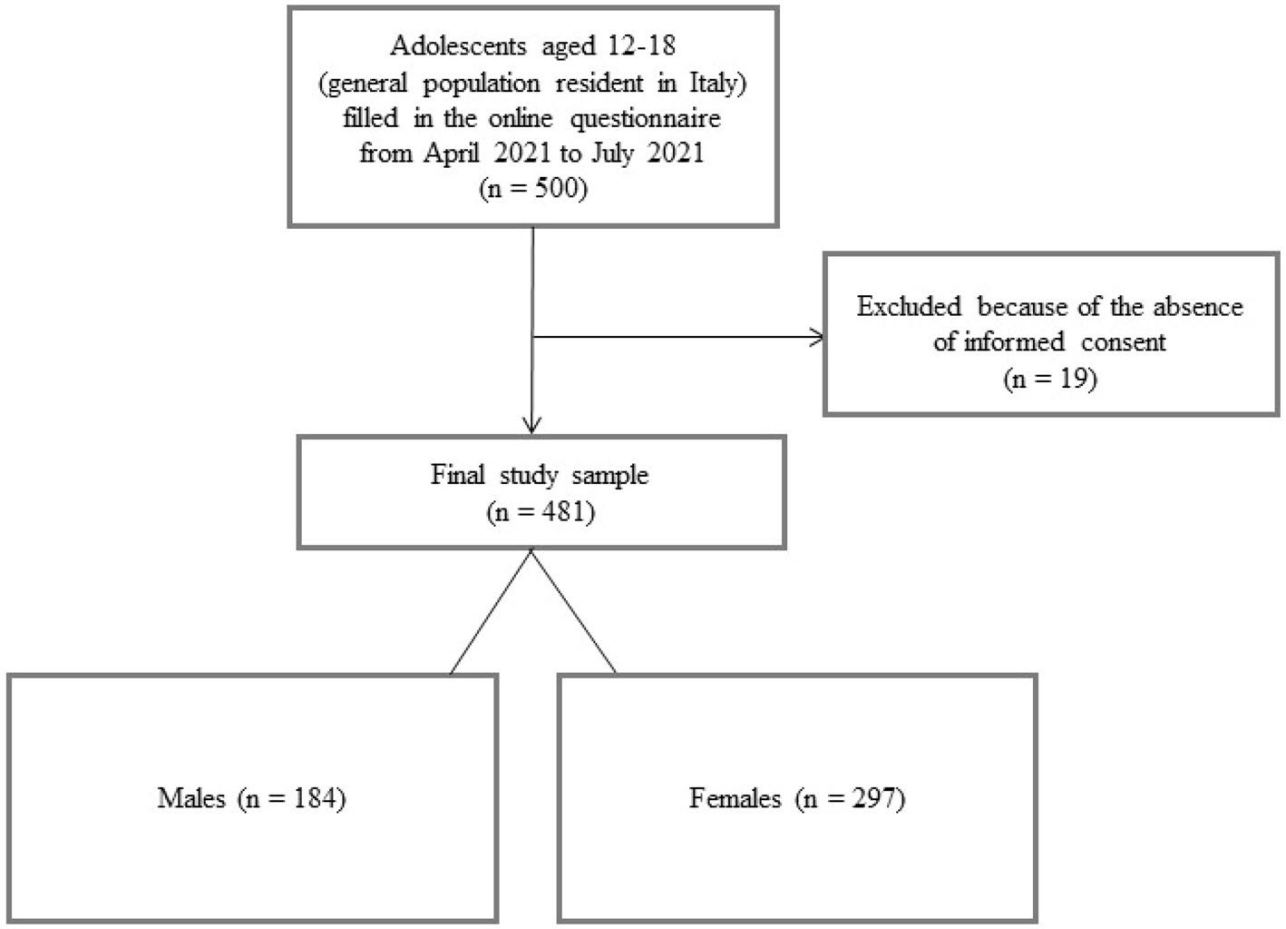
Flow chart of the study sample.

We enrolled adolescents aged 12–18 years resident in Italy, who voluntarily responded to an online survey diffused on social networks (Facebook, Instagram, and WhatsApp) and on the Institute's website from April 2021 to July 2021. In total, 500 participants from 13 Italian regions took part in the survey. We excluded all adolescents who refused to give written informed consent. Figure 1 shows the flow chart of the study sample.

### Procedures

All participants underwent a comprehensive assessment evaluating psychiatric symptoms using a questionnaire constructed using and integrating questions of diagnostic interviews validated in literature and scales to measure subjects’ level of functioning. This consisted of 56 items divided into three sections (also an English translation of the questionnaire is readable in Supplementary materials).

The first one collected personal information (year of birth, gender, region of residence, personal history of COVID-19), as well as whether the subject was undergoing psychological therapy, psychotherapy, or neuropsychiatric visits before the COVID-19 emergency, or during the pandemic. The second section detected the presence of stress disorders symptoms related to the current restriction measures and adolescents’ perception of stress level [e.g., “Are you currently feeling more or less stressed compared to the first wave of COVID-19 diffusion (March–May 2020)?”, “Do you often have unpleasant dreams or nightmares whose contents or emotions had to do with COVID-19? Never, occasionally, or often?”, “Do you still experience positive feelings (e.g., love, happiness, satisfaction)? Never, occasionally, or often?”]. The third section investigated the presence and the frequency of psychiatric symptoms to have a complete overview of the effect of the COVID-19 pandemic on adolescents [e.g., “In the past 2 weeks have you felt that you were very sad, down in the dumps, empty, or have you felt more often that you felt like crying? (Yes, 4 or more days a week, Yes, less than 4 days a week, No)”, “Have you ever had the feeling of sudden, uncontrollable fear associated with symptoms of physical discomfort (panic attack) in the recent period?”, “Does your appetite seem different in this last period?”, “Have you felt so bad in this last period that you thought of voluntarily harming yourself?”]. In particular, we integrated questions from two different scales, routinely administered to adolescent patients in Child Neuropsychiatry Units:

- K-SADS-PL DSM-5: the Kiddie—Schedule for Affective Disorders and Schizophrenia for School-Aged Children—Present and Lifetime Version is an interview for the evaluation of psychopathological disorders in children and adolescents^31^, to estimate stress and emotional disorders. This semi-structured interview based on DSM-5 diagnostic criteria allows detecting the presence of symptoms attributable to different psychiatric diseases such as social anxiety, generalized anxiety, panic attacks, mood disorders, anticonservative and self-harm thinking, social withdrawal, eating disorders, and sleep problems. We used K-SADS questions and cut-offs to determine subthreshold and suprathreshold symptoms. In detail, we used K-SADS questions to write items 38 and 39 asking whether adolescents had experienced panic attacks, how often (none, one, more than one), and whether they feared that they might recur. We assigned a subthreshold score if they positively answered to the first question, but negatively to the second one, and a suprathreshold score if they answered positively to both questions. We adopted the same scoring for anxiety symptoms (items 40 and 41). Moreover, to investigate depressive symptoms (items 42–45), and to determine whether symptoms were absent, subthreshold, or supra-threshold, we gave response options indicating the frequency of that symptom, as per DSM-5 criteria [not present; present but less than 4 days per week (subthreshold); present 4 or more days per week (suprathreshold)]. To explore eating symptoms we used item 46 (Does your appetite seem different in this last period?) asking any variation without setting a cut-off (e.g., Yes, I have less appetite, but my weight has not changed; Yes, I have less appetite and my weight has reduced significantly; etc.). We did the same with the sleep-related item (47), asking any differences in their sleep, and with suicidal ideation item (48) and self-harming one (49).
- CGAS—Children’s Global Assessment Scale^32^ to test the level of functional impairment taking into account the psychosocial and work/school functioning of the subject by assigning a score in a continuum ranging from mental health to very serious mental disorder, regardless of the nature of the psychiatric disorder. We used the 10 functioning ranges of the CGAS scale to ask adolescents whether the problems they were reporting in the questionnaire were in their opinion affecting their lives and to what extent [item 37 “Are these difficulties affecting your life?”]. We considered a relevant severity score if the participant gave a response from 4 included and above (“SOME PROBLEMS; those who know me really well may be concerned about me”). All symptoms with nonimpaired functioning (responses to item 37 from 3 and below) were considered neither subthreshold nor suprathreshold.

Last items aimed to understand how adolescents’ functioning has changed in response to the limited personal freedom [“Since the COVID-19 pandemic has spread, do you find that you feel less of a wish to meet your friends and feel a sense of “detachment” from them?”], and what kind of interventions they think adults should implement [“What do you think adults could do to help adolescents as you?”].

### Statistical analysis

Statistical analyses were performed with the Statistical Package for Social Science (SPSS 27.0) for Mac. We first run descriptive analyses, including mean values and standard deviation (SD), as appropriate for continuous variables (i.e., age), and absolute and relative frequencies for categorical variables. We then compared demographic and clinical variables between genders, using the Chi-square test, complemented by post-hoc analyses. All tests were two-sided, with the alpha set at 0.05. All authors have complete access to our database, in which data was collected only after pseudonymization.

### Ethics approval and consent to participate

The study received the approval of the Ethics Committee of Policlinico San Matteo in Pavia, Italy (P-20210060206). Every participant gave his/her written informed consent and was free to withdraw their participation in the study at any time.

## Results

### Study population

We included 481 adolescents (*M*_*age*_ = 15.04; SD = 1.93; 61.74% female) (Fig. 1). Table 1 shows sociodemographic data for the total sample and the two subgroups.

**Table 1:** Sociodemographic data in the total sample and the two subgroups.

Characteristic	Total (N = 481)	Males (N = 184)	Females- (N = 297)	*p*
**Sociodemographic data**				
Mean age (SD)	15.04 (1.93)	14.95 (1.88)	15.08 (1.95)	.467
Nation of birth, N (%)				.076
Italian	455 (94.6)	169 (91.85)	286 (96.29)	
European	12 (2.49)	8 (4.35)	4 (1.35)	
Other	14 (2.91)	7 (3.80)	7 (2.36)	
Region of residence, N (%)				.088
Campania	2 (0.42)	1 (0.54)	1 (0.34)	
Emilia-Romagna	5 (1.04)	3 (1.63)	2 (0.67)	
Lazio	4 (0.83)	2 (1.09)	2 (0.67)	
Liguria	2 (0.42)	0 (0)	2 (0.67)	
Lombardy	379 (78.78)	140 (76.09)	239 (80.47)	
Molise	1 (0.21)	1 (0.54)	0 (0)	
Piedmont	62 (12.89)	23 (12.50)	39 (13.13)	
Puglia	1 (0.21)	0 (0)	1 (0.34)	
Sardinia	16 (3.33)	12 (6.52)	4 (1.35)	
Sicily	2 (0.42)	0 (0)	2 (0.67)	
Tuscany	3 (0.62)	2 (1.09)	1 (0.34)	
Valle d’Aosta	1 (0.21)	0 (0)	1 (0.34)	
Veneto	3 (0.62)	0 (0)	3 (1.01)	
Adolescents who contracted COVID-19 disease, N (%)	65 (13.51)	27 (14.67)	38 (12.80)	.558
Therapy before pandemic	39 (8.11)	14 (7.61)	25 (8.42)	
Therapy continued in-person during pandemic	14 (2.91)	4 (2.17)	10 (3.37)	
Therapy continued online during pandemic	11 (2.29)	3 (1.63)	8 (2.69)	
Therapy started in-person during pandemic	18 (3.74)	5 (2.72)	13 (4.38)	
Therapy started online during pandemic	6 (1.25)	2 (1.09)	4 (1.35)	
Therapy stopped during pandemic	23 (4.78)	11 (5.98)	12 (4.04)	
Perceived Stress, mean (SD)	5.64 (3.11)	5.05 (3.32)	6.02 (2.91)	< .001**
Higher level of perceived stress compared to first lockdown, N (%)	199 (41.37)	60 (32.61)	139 (46.80)	
Lower level of perceived stress compared to first lockdown, N (%)	194 (40.33)	89 (48.37)	105 (35.35)	
Same level of stress perceived during the first lockdown, N (%)	88 (18.30)	35 (19.02)	53 (17.85)	
Trauma before pandemic	103 (21.41)	29 (15.76)	74 (24.92)	.017

Significance: * =  < .05; ** < .01.

### First outcome

We studied the prevalence rate of COVID‐19‐related stress disorders, and depressive, anxious, panic, psychotic symptoms, according to DSM-5 criteria. Results are reported in Table 2.

**Table 2:** Prevalence rate of psychopathological symptoms according to DSM-5 criteria.

	Total N = 378	Suprathreshold	Subthreshold
PTSD, N (%)	75 (19.84)	7 (1.85)	68 (17.99)
ASD, N (%)	11 (2.91)	–	–

	Total N = 481	Suprathreshold	Subthreshold
During pandemic psychotic symptoms ^a^, N (%)	77 (16.01)	–	–
Pre-pandemic psychotic symptoms ^a^, N (%)	58 (12.06)	–	–
Anxiety, N (%)	285 (59.25)	225 (46.78)	60 (12.47)
Panic, N (%)	188 (39.09)	121 (25.16)	67 (13.93)
Depression, N (%)	254 (52.80)	90 (18.71)	164 (34.09)

^a^Not due to substance use.

**Figure 2:**
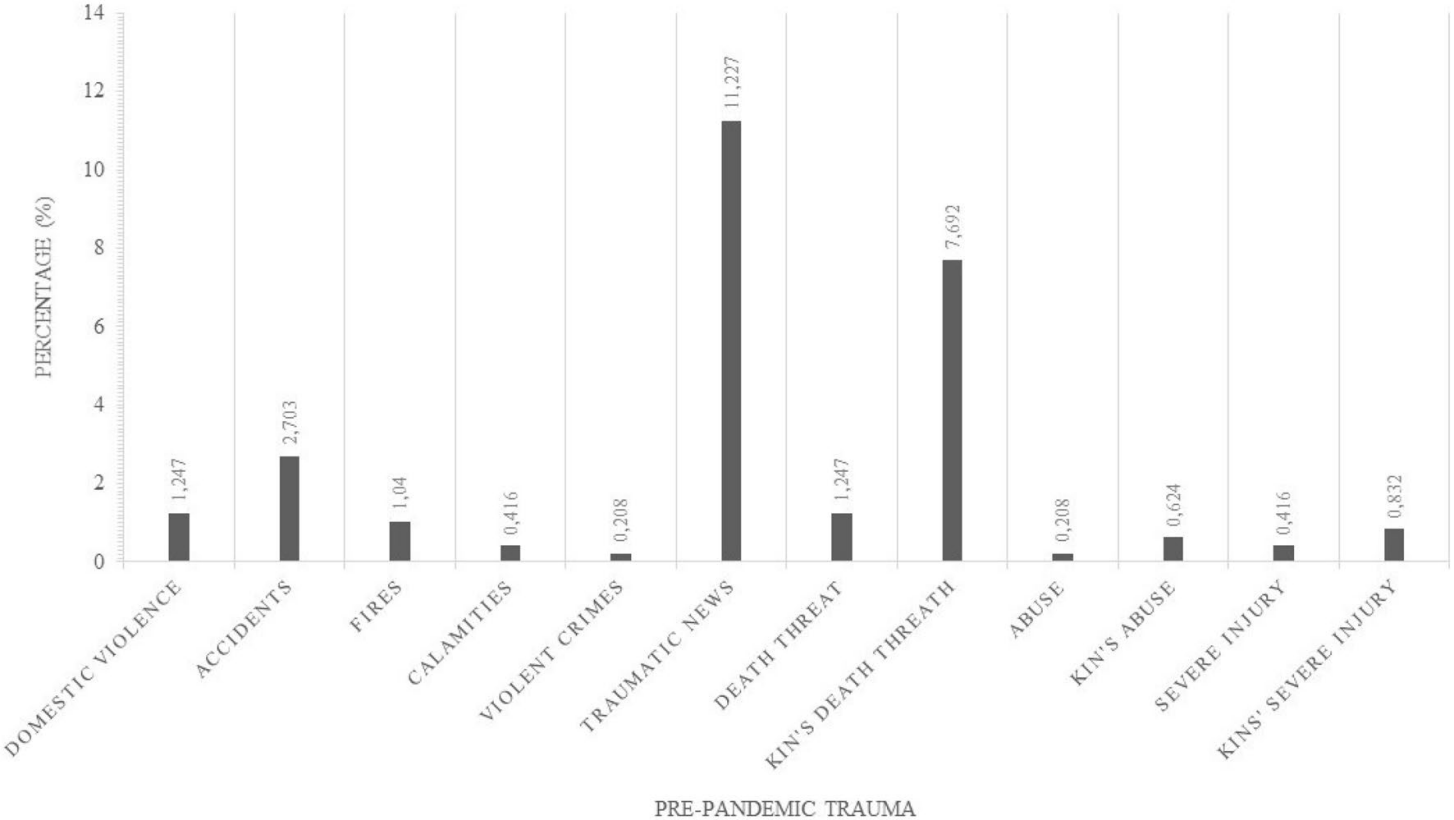
Pre-pandemic trauma reported.

Regarding stress disorders, to minimize biases, we excluded 103 adolescents who reported any trauma before COVID-19 pandemic (Fig. 2, Table S1).

### Second outcome

Table 3 shows the prevalence rate of dysfunctional eating behaviors, sleep problems, substances abuse, social withdrawal, anti-conservative, suicidal ideation, and self-harm in the total sample Perceived personal stress level is reported in Table 1.

**Table 3:** Prevalence rate of dysfunctional eating behaviors, sleep problems, substances abuse, and social withdrawal.

		N (%)
Changing in eating behavior	No changes	233 (48.44)
	Less appetite with unchanged weight	107 (22.24)
	Less appetite with reduced weight	31 (6.44)
	More appetite with unchanged weight	66 (13.72)
	More appetite with increased weight	44 (9.14)
Sleep disorders	No changes	207 (43.03)
	Difficulty falling asleep	87 (18.08)
	Night awakenings	63 (13.09)
	Early awakenings	38 (7.90)
	Daytime naps	86 (17.87)
Frequency of anti-conservative and suicidal ideation	Never	337 (70.06)
	Sometimes	104 (21.2)
	Often	40 (8.32)
Non-suicidal self-harm	Never	357 (74.22)
	Just the thought of self-harming	80 (16.63)
	Minor self-harming gestures	43 (8.94)
	Severe self-harming gestures	1 (0.20)
Regular use of drugs/alcohol in the last 6 months	No	452 (93.97)
	Yes	29 (6.03)
Withdrawal perceived	Before pandemic	63 (13.10)
	During pandemic	73 (15.18)

### Third outcome

We also compared males’ and females’ prevalence of symptoms. Table 4 shows comparisons between genres.

**Table 4:** Comparison between gender.

	Males (N = 184) N (%)	Females- (N = 297) N (%)	χ^2^	*p*		
APT +	14 (7.61)	35 (11.79)	2.16	.141		
ASD	0 (0)	11 (3.70)	7.87	.005**		
Pre-pandemic psychotic symptoms ^a^	13 (7.07)	45 (15.15)	7.01	.008**		
Post-pandemic psychotic symptoms ^a^	24 (13.04)	53 (17.85)	1.948	.163		
Suicidal ideation	32 (17.39)	112 (37.71)	22.36	< .001**		
Non-suicidal self-harm	27 (14.67)	97 (32.66)	19.21	< .001**		
Withdrawal perceived before pandemic	26 (14.13)	37 (12.46)	0.279	.597		
Withdrawal perceived during the pandemic	21 (11.41)	52 (17.51)	3.279	.07		
Any change in eating behavior	66 (35.87)	182 (61.28)	38.53	< .001**		
Any sleep disorder	73 (39.67)	201 (67.68)	39.45	< .001**		
Regular use of drugs/alcohol in the last 6 months	12 (6.52)	17 (5.72)	.128	.721		
Psychotic symptoms associated with substance use (pre-pandemic)	0 (0)	7 (2.36)	4.401	0.036*		
Psychotic symptoms associated with substance use (during pandemic)	0 (0)	3 (1.01)	1.87	0.171		

	Males (N = 184) N (%)		Females- (N = 297) N (%)		χ^2^	*p*
	Supra	Sub	Supra	Sub		
PTSD	3 (1.63)	17 (9.24)	4 (1.35)	51 (17.17)	8.80	.012*
Anxiety	53 (28.80)	27 (14.67)	172 (57.91)	33 (11.11)	39.93	< .001**
Depression	16 (8.70)	47 (25.54)	74 (24.92)	117 (39.39)	44.14	< .001**
Panic	23 (12.50)	14 (7.61)	98 (33.00)	53 (17.85)	45.14	< .001**

Significance: * ≤ 05 ; **  ≤ .01.

^a^Not due to substance use.

## Discussion

The literature showed how the restrictive measures taken due to the ongoing health emergency have affected several emotional and psychological areas^3,7–9^. In continuity with a previous study^28^, we used an online survey to evaluate the psychological and emotional impact of the COVID-19 pandemic on adolescents during the third lockdown in Italy (March–June 2021). We enrolled 481 participants that do not differ in gender, nation of birth, region of residence, and personal experience of virus infection. In the sample, almost 8% stated that were already in therapy before the pandemic, with half of them continuing it, and 5% claim to have started treatment during the COVID-19 health emergency. This data confirms how the pandemic and quarantine influenced psychological health and the need for greater support for Italian adolescents ^9^.

As the first outcome, we aimed to find the prevalence rate of depressive, anxious, panic, and psychotic symptoms and COVID-related stress disorders, according to DSM-5 criteria, during the third lockdown in Italy. We found that adolescents reported feeling that they experienced a higher level of psychotic symptoms (not due to substance abuse) than in the pre-pandemic period (+ 4%). In line with the literature^1,2^, the survey also revealed that more than half of the population presented anxiety symptoms, with almost 47% of them with suprathreshold symptoms. About panic, we found that 39% of adolescents experienced panic symptoms, 25% of them with suprathreshold severity. Regarding depression, results showed that 34% exhibited subthreshold symptoms and almost 19% suprathreshold. In contrast to some studies showing a prevalence of depressive disorders, followed by anxiety ones^8,10^ our study shows a higher frequency of anxiety disorders than depressive disorders, in fact, unpredictability, the climate of uncertainty, and general instability appear to be fertile substrates for the onset of anxiety disorders. Finally, we aimed to evaluate the prevalence of COVID-related stress disorders, excluding those who had experienced previous trauma. Among them, traumatic news and abuse of a friend represented the most frequent traumas, and some of the participants reported multiple experienced traumas. In the rest of the sample, we discovered that almost 20% presented stress symptoms: seven adolescents (1.85%) met the criteria for a PTSD diagnosis according to DSM-5 and 11 participants (2.91%) met the criteria for ASD. Moreover, 18% experienced subthreshold PTSD symptoms. These data are alarming when compared to an earlier survey in which only 2 out of 1251 (0.16%) participants met the criteria for a PTSD diagnosis and 1 (0.08%) for ASD^28^. The increase in PTSD and ASD diagnoses may be due to the continuation of restrictive measure. The reduction of social relations, the interruption of routines, social isolation, distancing, and other restrictive measures adopted are therefore confirmed as precipitating factors for the onset of stress disorders^33^. Moreover, also school closures and e-learning affected adolescents’ health in fact, not only did they suffer from the lack of classmates, but they also suffered from the considerable study load required.

As the second objective, we investigated the perception of personal stress level, sleep problems, dysfunctional eating behaviors, substances abuse, perceived social withdrawal, and suicidal ideation and self-harm during the third lockdown in Italy. We noticed that 41% of the sample indicated that they had a perception that their stress levels had increased since the first lockdown, and literature confirms that stress levels follow a trend, increasing during the closing phases and decreasing near re-openings^3^. Moreover, it is interesting to learn that more than half of adolescents reported sleep disorders, especially difficulty falling asleep, night awakenings, and daytime naps. As shown by the studies of Uccella and colleagues^9^ on the Italian population during the pandemic, sleep disorders occupy a significant place both in the adolescent population and in children under 6 years of age. Significant changes were also reported in eating disorders. More than half of adolescents manifested changes in eating behavior, and a relevant percentage reported variations in body weight. In this regard, Rodgers and colleagues^17^ described some important precipitating factors such as limited access to support networks, increased symptoms of self-evaluation about one's body due to social isolation, and experiences of anxiety related to food, exercise, and weight due to the influence of social media. We also found that 6% of adolescents that reported regular use of drugs and/or alcohol in the last 6 months. Furthermore, the study sample reported a higher level of withdrawal symptoms during the pandemic compared to the pre-COVID period (+ 15%). This finding overlaps with the literature stating that withdrawal was likely used during the pandemic as a strategy to reduce perceived psychological stress^20^. Lamblin and colleagues^26^ then found that interactions between brain maturation and external stimuli might increase the risk of mental illness or promote resilience. A great concern is also aroused by the presence of active suicidal ideation (almost 30%), higher than in literature^18^. Our findings confirm what Hill and colleagues^15^ said about the alarming increase in the discomfort experienced by adolescents during the pandemic. Moreover, also non-suicidal self-harm ideations and acts such as cutting, burning, and branding must be considered as an “alert bell” among adolescents (25.7%) with even 9% of adolescents reporting actual mild or severe self-harm gestures.

For third aim, in line with the literature^10,18,19^, we found that female participants exhibited a higher frequency of anxiety (57% suprathreshold), panic (33% suprathreshold), and depressive symptoms (almost 25% suprathreshold and 39% subthreshold). Moreover, male adolescents showed fewer pre-pandemic psychotic symptoms (either due to drugs or not) than females, but they do not differ in regular substance use. Female adolescents also experienced more symptoms of ASD and PTSD than males and expressed a higher level of suicidal ideation (37%) and non-suicidal self-harm (almost 33%). Then, in line with previous data^34,35^, we found a higher level of expressed sleep problems and problems related to eating behavior in the female population. The prevalence of psychiatric symptoms in the female population is not unambiguous, however these data would seem to reflect a higher prevalence of symptoms such as anxiety, depression and eating disorders in females. Consistent with other studies, psychological symptoms were found to be significantly more common among females, and this finding is not surprising because, regardless of the pandemic, anxiety and depressive symptoms are generally more common in females, even in adolescence^36^.

Regarding limitations, the online survey was accessible from April to July 2021; therefore, the brief period that coincides with the final phase of the third lockdown in Italy did not encourage compilation to a greater number of adolescents, but to limit biases, we still decided to stop data collection before the end of restrictions. Moreover, we cannot generalize the data to the entire Italian adolescent population because the answers to the questionnaire came mainly from northern Italy, and only adolescents who had technological devices and an internet connection were able to access the survey.

Since the high frequency and severity of symptoms, it appears necessary to ensure valid and continuous assistance and not to overlook the signs of this “latent pandemic” that involves above all the most fragile and emotionally vulnerable in a critical growth phase, that of adolescence. The increased frequency of symptoms associated with various psychiatric disorders triggered by the ongoing pandemic has inevitably resulted in a significant impact on clinical practice. Several epidemiological investigations have established a disproportionate increase in admissions to child neuropsychiatry wards and an increase in emergencies. In our opinion, it is therefore essential to provide appropriate and prompt conditions of care, maybe creating networks among services to intercept distress at an early stage. Moreover, as other studies suggested, rather than talking about “social distancing”, measures should be planned to allow “physical distancing” while preserving “social connection”^37^. Therefore, it is necessary to listen to adolescents and accept their discomfort, taking time to talk about their emotional well-being, especially under the most difficult conditions and in the contexts in which they spend the most time, such as school. To some extent, telemedicine has facilitated access to care, but the demand for care from young people is still very strong and resources need to be used to provide better care and recognize psychological and social distress. To end, in the final part of our questionnaire, we asked young people to express themselves freely and give their suggestions on what changes adults should make to the current level of care. In several comments, the adolescents asked adults to give them a space to listen, to include them more, and make them more responsible, but at the same time leave them a space for physiological growth and search for independence (Figure S1).

## Supplementary Information

Supplementary Figure S1.

Supplementary Table S1.

Supplementary Information 3.

Supplementary Information 4.

Supplementary Information 5.

## Data Availability

The dataset generated and analyzed during the current study is available upon request in the Zenodo repository^30^ at 10.5281/zenodo.5746605.
