# Supplementary figures and images for: Psychiatric symptoms and emotional impact of the COVID-19 pandemic on Italian adolescents during the third lockdown: a cross-sectional cohort study
Source: Sci Rep. 2022 Dec 3;12:20901. doi: 10.1038/s41598-022-25358-0 (PMC9719459; doi:10.1038/s41598-022-25358-0)

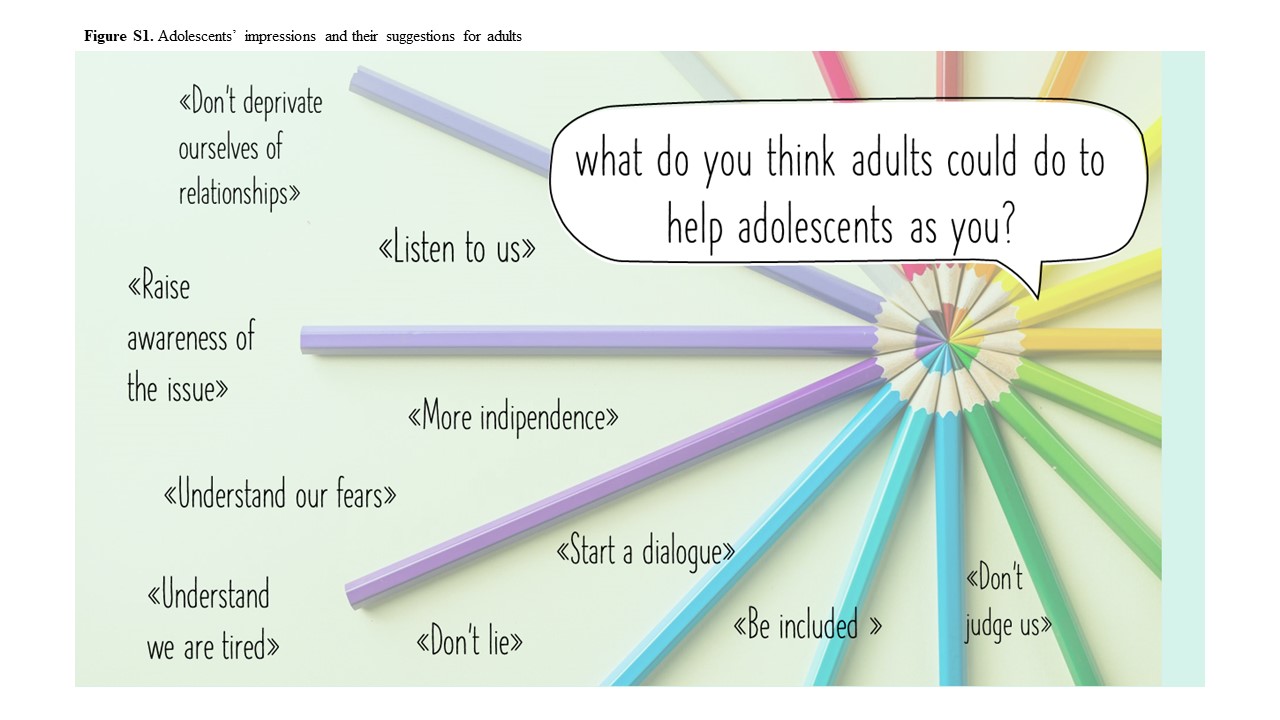

Supplement: Supplementary file 1 — Supplementary Figure S1. [file 41598_2022_25358_MOESM1_ESM.jpg]
