# Supplementary material for: Psychiatric symptoms and emotional impact of the COVID-19 pandemic on Italian adolescents during the third lockdown: a cross-sectional cohort study
Source: Sci Rep. 2022 Dec 3;12:20901. doi: 10.1038/s41598-022-25358-0 (PMC9719459; doi:10.1038/s41598-022-25358-0)
Supplement: Supplementary file 2 — Supplementary Table S1. [file 41598_2022_25358_MOESM2_ESM.docx]

**Table S1**

*Sociodemographic data of the 103 adolescents who reported previous trauma before COVID-19 pandemic*

| **Characteristic** | **Total (N=103)** |
| --- | --- |
| ***Sociodemographic data*** | **N (%)** |
| Year of birth |  |
| *2003* | 28 (27.18) |
| *2004* | 15 (14.56) |
| *2005* | 17 (16.51) |
| *2006* | 7 (6.80) |
| *2007* | 25 (24.27) |
| *2008* | 6 (5.83) |
| *2009* | 5 (4.85) |
| Sex, female | 74 (71.85) |
| Region of Residence |  |
| *Campania* | 1 (0.97) |
| *Liguria* | 1 (0.97) |
| *Lombardy* | 75 (72.82) |
| *Molise* | 1 (0.97) |
| *Piedmont* | 20 (19.42) |
| *Sardinia* | 1 (0.97) |
| *Tuscany* | 1 (0.97) |
| *Valle d’Aosta* | 1 (0.97) |
| *Veneto* | 2 (1.94) |
| Psychological therapy and/or neuropsychiatric visits before COVID-19 pandemic | 15 (14.56) |
