# Supplementary material for: Psychiatric symptoms and emotional impact of the COVID-19 pandemic on Italian adolescents during the third lockdown: a cross-sectional cohort study
Source: Sci Rep. 2022 Dec 3;12:20901. doi: 10.1038/s41598-022-25358-0 (PMC9719459; doi:10.1038/s41598-022-25358-0)
Supplement: Supplementary file 3 — Supplementary Information 3. [file 41598_2022_25358_MOESM3_ESM.pdf]

# The emotional impact of COVID-19 on adolescents in Italy - 2021

Hello!
Anti-contagion provisions have disrupted everyone's lifestyle and pace of life, particularly impacting adolescents.
We are asking for your help in trying to understand the best way through which we adults, who have been working with adolescent-related distress and difficulties for years, could lend a hand to you or to some of your peers who are experiencing difficult times related to the current pandemic.
For this very reason, we are asking you to answer some questions in which the emotional impact that the current health emergency is having on young people aged 12-18 will be explored.
Afterwards, if you wish, we ask you to also spread the link among your friends and acquaintances to help us collect as many questionnaires as possible with respect to Italian adolescents.

If you agree to participate in this study, we will ask you to fill out a short online questionnaire ENTIRELY ANONYMOUS created to assess the impact of restrictive measures on your life and health.
You will need about 5 minutes to complete it.
Your participation in the study is completely voluntary and poses no risk to your health.
Should you feel uncomfortable answering any question, you can stop the survey at any time without any problem.

The questionnaire is anonymous and complies with all research ethical standards regarding the protection of personal data, which is protected both by the specific legislation (DL 101/18) and by the research project managers.
The developer of this study is the Istituto di Ricovero e Cura a Carattere Scientifico, Casimiro Mondino National Neurological Institute Foundation of Pavia.
If you would like to receive information regarding privacy regulations and ask us any questions, you can send an email to this address: covid19.ado@mondino.it
We will get back to you as soon as possible.

Thank you for your time and cooperation!

---

* Mandatory

1. Informed Consent *

   I consent to the collection of Personal Data for the purposes stated in the policy.

   *Mark only one answer.*

   - ◯ I agree and continue filling out the questionnaire
   - ◯ I do not consent and do not start filling out the questionnaire

Personal data

2. Create a nickname *

3. What is your year of birth? *

*Mark only one answer.*

- 2003
- 2004
- 2005
- 2006
- 2007
- 2008
- 2009

4. Gender *

*Mark only one answer.*

- Female
- Male

5. Country of birth *

*Mark only one answer.*

- Italy
- Abroad (Europe)
- Abroad (other continent)

6. Italian region where you live *

*Mark only one answer.*

- Abruzzo
- Basilicata
- Calabria
- Campania
- Emilia-Romagna
- Friuli-Venezia Giulia
- Lazio
- Liguria
- Lombardy
- Marche
- Molise
- Piedmont
- Puglia
- Sardinia
- Sicily
- Tuscany
- Trentino-Alto Adige
- Umbria
- Valle d'Aosta
- Veneto

7. Prior to the COVID-19 emergency, were you undergoing psychological therapy, psychotherapy, and/or neuropsychiatric visits? *

*Select all applicable items.*

- [ ] No
- [ ] Psychological therapy
- [ ] Psychotherapy
- [ ] Neuropsychiatric visits

8. During the months of restrictions did you begin or continue psychological therapy, psychotherapy, and/or neuropsychiatric visits? *

*Mark only one answer.*

- ( ) Yes, I continued the sessions mainly in presence
- ( ) Yes, I continued the sessions mainly in the online mode
- ( ) Yes, I started psychological therapy/ neuropsychiatric visits in presence
- ( ) Yes, I started psychological therapy/neuropsychiatric visits in online mode
- ( ) No, during the pandemic I stopped psychological therapy/neuropsychiatric visits
- ( ) I was not and am not receiving any kind of therapy

9. In the past 6 months, have you personally experienced or witnessed traumatic events NOT related in any way to the COVID-19 pandemic? *

*Select all applicable items.*

- [ ] No
- [ ] Domestic violence
- [ ] Accidents
- [ ] Fires
- [ ] Calamities
- [ ] Violent crimes
- [ ] Traumatic news
- [ ] Death threat
- [ ] Kin's death threat
- [ ] Abuse
- [ ] Kin's abuse
- [ ] Severe injury
- [ ] Kin's severe injury

10. Have you contracted COVID-19 disease? *

*Mark only one answer.*

- ( ) No
- ( ) Yes

11. If yes, when did you contract the disease?

*Mark only one answer.*

- November 2019
- December 2019
- January 2020
- February 2020
- March 2020
- April 2020
- May 2020
- June 2020
- July 2020
- August 2020
- September 2020
- October 2020
- November 2020
- December 2020
- January 2021
- February 2021
- March 2021
- April 2021
- Other: ______________

12. Currently, how stressed do you feel because of the restrictions? *

*Mark only one answer.*

| Not at all | |
|---|---|
| 0 | ◯ |
| 1 | ◯ |
| 2 | ◯ |
| 3 | ◯ |
| 4 | ◯ |
| 5 | ◯ |
| 6 | ◯ |
| 7 | ◯ |
| 8 | ◯ |
| 9 | ◯ |
| 10 | ◯ |
| Very much | |

13. If you feel stressed, what causes you the most stress?

______________________________

14. Are you currently feeling more or less stressed compared to the first wave of COVID-19 diffusion (March-May 2020)? *

*Mark only one answer.*

- More stressed than before
- Less stressed than before
- I feel a level of stress equal to that experienced at that time

15. Do you often have unpleasant and/or negative thoughts with respect to the COVID-19 pandemic that you can't avoid thinking about? Never, occasionally, or often? *

*Mark only one answer.*

| | |
|---|---|
| | Never |
| 0 | ◯ |
| 1 | ◯ |
| 2 | ◯ |
| | Often |

16. Do you often have unpleasant dreams or nightmares whose contents or emotions had to do with COVID-19? Never, occasionally or often? *

*Mark only one answer.*

| | Never |
|---|---|
| 0 | ◯ |
| 1 | ◯ |
| 2 | ◯ |
| | Often |

17. Do you experience the unpleasant events caused by COVID-19 days after the event as if they were happening to you again at that time (e.g., flashbacks)? Never, occasionally, or often? *

*Mark only one answer.*

| | Never |
|---|---|
| 0 | ◯ |
| 1 | ◯ |
| 2 | ◯ |
| | Often |

18. If something happens that reminds you of the incident you were involved in because of COVID-19 do you experience intense psychological distress? Never, occasionally, or often? *

*Mark only one answer.*

| | Never |
|---|---|
| 0 | |
| 1 | |
| 2 | |
| | Often |

19. Do you still feel able to feel positive feelings (e.g., love, happiness, satisfaction)? Never, occasionally or often? *

*Mark only one answer.*

| | Never |
|---|---|
| 0 | |
| 1 | |
| 2 | |
| | Often |

20. If something happens that reminds you of the episode you were involved in because of COVID-19, do you manifest exaggerated alarm reactions? (e.g., increased heart rate, difficulty breathing) *

*Mark only one answer.*

- Yes
- No

21. Do you ever have the feeling of being unreal, as if you were on the outside of your own body? Of feeling confused with respect to the passage of time? Never, occasionally or often? *

*Mark only one answer.*

| | |
|---|---|
| | Never |
| 0 | |
| 1 | |
| 2 | |
| | Often |

22. If you answered the previous question occasionally or often, were these feelings present even before the COVID-19 pandemic?

*Mark only one answer.*

- Yes
- No

23. Do you feel that some memories of what happened because of COVID-19 have faded from your mind or are there parts or details that you cannot remember? Never, occasionally, or often? *

*Mark only one answer.*

| | |
|---|---|
| | Never |
| 0 | ◯ |
| 1 | ◯ |
| 2 | ◯ |
| | Often |

24. Since the COVID-19 pandemic spread, do you find yourself thinking that you are bad, or that no one can be trusted, or that the world is absolutely dangerous? *

*Mark only one answer.*

◯ Yes
◯ No

25. Do you happen to blame yourself or others for what is happening (pandemic COVID-19)? *

*Mark only one answer.*

◯ Yes
◯ No

26. Since the spread of the COVID-19 pandemic, do you find that you always feel angry, afraid, guilty? *

*Mark only one answer.*

- Yes
- No

27. Do you try to avoid unpleasant memories, thoughts, or feelings related to or associated with what happened to you because of COVID-19? Never, occasionally, or often? *

*Mark only one answer.*

Yes

0

1

2

Often

28. Do you try to avoid people, places, conversations, activities, objects, situations that activate memories or thoughts related to COVID-19? Never, occasionally or often? *

*Mark only one answer.*

| |
|---|
| Never |
| 0 ⬭ |
| 1 ⬭ |
| 2 ⬭ |
| Often |

29. Do you feel more sensitive than in the past to potential threats? Never, occasionally, or often? *

*Mark only one answer.*

| |
|---|
| Never |
| 0 ⬭ |
| 1 ⬭ |
| 2 ⬭ |
| Often |

30. Do you have difficulty keeping in mind what you are doing? Do you find it more difficult to do homework or activities that you find fun since COVID-19 became widespread? Never, occasionally, or often? *

*Mark only one answer.*

Never

0 ○

1 ○

2 ○

Often

31. Do you feel that you are more nervous? For example, does even a cough or a sneeze scare you so much? Never, occasionally, or often? *

*Mark only one answer.*

Never

0 ○

1 ○

2 ○

Often

32. Since the COVID-19 pandemic began, do you engage in reckless or self-destructive behaviors? *

*Mark only one answer.*

- Yes
- No

33. Have you experienced, in the past 6 months, that your imagination plays tricks on you, such as seeing or hearing things that others do not seem to perceive and that disorient you (e.g., visions)? Never, occasionally, or often? *

*Mark only one answer.*

| | |
|---|---|
| | Never |
| 0 | ⬭ |
| 1 | ⬭ |
| 2 | ⬭ |
| | Often |

34. If you answered the previous question occasionally or often: were these feelings present even before the COVID-19 pandemic?

*Mark only one answer.*

- Yes
- No

35. Have you used drugs or alcohol habitually (e.g., 2-3 times a week) in the past 6 months? *

*Mark only one answer.*

- Yes
- No

36. The difficulties that you answered OFTEN in the previous questions, how long have they been present?

*Mark only one answer.*

- From 1 to 3 days
- From 4 to 30 days
- More than 30 days

37. Are these difficulties affecting your life?

*Mark only one answer.*

- DOING VERY WELL; I have no problems at home or with friends; I participate in activities and have interests.
- DOING WELL; safe at home or with friends but I rarely get mildly agitated.
- DOING ALL RIGHT; safe at home or with friends but I become agitated as a result of a stressful situation
- SOME PROBLEMS; those who know me really well may be concerned about me
- SOME NOTICEABLE PROBLEMS; problems are obvious to all but only in some situations
- OBVIOUS PROBLEMS; several problems that make me feel bad in most situations
- SERIOUS PROBLEMS; I am very sick and go into crisis in some situations
- SEVERE PROBLEMS; I'm very sick and I'm always in crisis
- VERY SEVERELY IMPAIRED; I am very sick and I need someone to help me and be with me
- EXTREMELY IMPAIRED; sono così malato che ho bisogno di una supervisione costante

38. Have you ever had the feeling of sudden, uncontrollable fear associated with symptoms of physical discomfort (panic attack) in the recent period? *

*Mark only one answer.*

- Yes, once
- Yes, more than once
- No

39. If yes, have you ever felt that because of panic attacks you avoided doing something or were afraid that the attacks would recur?

*Mark only one answer.*

- Yes
- No

40. In the past 6 months have you been worrying excessively about many things, perhaps more than your peers, without being able to control this worry? *

*Mark only one answer.*

- Yes
- No

41. If so, have you experienced anxiety and worry being associated with symptoms such as tension, fatigue, concentration and memory problems, irritability, and sleep alterations?

*Mark only one answer.*

- Yes
- No

42. In the past 2 weeks have you felt that you were very sad, down in the dumps, empty, or have you felt more often that you felt like crying? *

*Mark only one answer.*

- Yes, 4 or more days a week
- Yes, less than 4 days a week
- No

43. In the last 2 weeks have you felt that you are very listless, without energy, and/or particularly bored? Do you feel that the things you used to enjoy before now you no longer enjoy or enjoy less than before? *

*Mark only one answer.*

- Yes, 4 or more days a week
- Yes, less than 4 days a week
- No

44. In the past 2 weeks, have you found it difficult to concentrate and pay attention to things? *

*Mark only one answer.*

- Yes, 4 or more days a week
- Yes, less than 4 days a week
- No

45. In the last 2 weeks have you felt restless or, on the contrary, slowed down? *

*Mark only one answer.*

- Yes, 4 or more days a week
- Yes, less than 4 days a week
- No

46. Does your appetite seem different in this last period? *

*Mark only one answer.*

- No
- Yes, I have less appetite but my weight has not changed
- Yes, I have less appetite and my weight has greatly reduced
- Yes, I have more appetite but my weight has not changed
- Yes, I have more appetite and my weight has significantly increased

47. At this time what is your sleep like most days? *

*Mark only one answer.*

- I sleep regularly at night and feel rested
- I don't go to sleep late, but it takes me more than an hour to fall asleepI
- often wake up during the night and struggle to fall back asleep
- I wake up early in the morning and never go back to sleep
- I don't get enough sleep at night and take naps (even hours) during the day

48. Have you ever thought in this last period that it would be better to die? *

*Mark only one answer.*

- Never
- Yes, occasionally
- Yes, often

49. Have you felt so bad in this last period that you thought of deliberately hurting yourself? *

*Mark only one answer.*

- No
- Yes, I just thought that
- Yes, I hurt myself but without serious medical consequences
- Yes, I hurt myself with serious medical consequences (e.g., needing hospitalization, ER, stitches...)

50. Have you experienced excessive verbal and/or physical outbursts of anger in this past year? *

*Mark only one answer.*

- No
- Yes, occasionally
- Yes, every day

51. Since the COVID-19 pandemic spread, do you find that you feel less of a desire to meet your friends and feel a sense of "detachment" from them? *

*Mark only one answer.*

- Yes
- No

52. If the restrictive measures preventing going out were not in place, would you prefer to go out or stay home anyway? *

*Mark only one answer.*

- Go out
- Stay home

53. Before the COVID-19 emergency, what did you prefer? *

*Mark only one answer.*

- Go out
- Stay home

54. Now that the restrictive measures have been eased you feel... (more than one possible answer) *

*Select all applicable items.*

- [ ] Happy because you can't wait to go out and meet friends/other people
- [ ] Scared because you don't think you are ready to go out and meet friends/other people
- [ ] Fearful about the possibility of contracting COVID-19
- [ ] Concerned about going back to school

55. What do you think adults could do to help adolescents as you?
