# Supplementary material for: Psychiatric symptoms and emotional impact of the COVID-19 pandemic on Italian adolescents during the third lockdown: a cross-sectional cohort study
Source: Sci Rep. 2022 Dec 3;12:20901. doi: 10.1038/s41598-022-25358-0 (PMC9719459; doi:10.1038/s41598-022-25358-0)
Supplement: Supplementary file 5 — Supplementary Information 5. [file 41598_2022_25358_MOESM5_ESM.pdf]

L'impatto emotivo del COVID-19 sugli adolescenti in Italia - 2021

Ciao!
Le disposizioni anti-contagio hanno stravolto lo stile e i ritmi di vita di tutti, in modo particolare hanno avuto un impatto molto forte sugli adolescenti.
Chiediamo il tuo aiuto per cercare di comprendere il modo migliore attraverso cui noi adulti, che da anni lavoriamo con il disagio e le difficoltà legate all'adolescenza, potremmo dare una mano a te o a qualche tuo/a coetaneo/a che sta vivendo dei momenti di difficoltà legati alla pandemia in corso.
Proprio per questo, ti chiediamo di rispondere ad alcune domande in cui verrà approfondito l'impatto emotivo che l'emergenza sanitaria in atto sta provocando sui giovani d'età compresa tra i 12 e i 18 anni.
Successivamente, se vorrai, ti chiediamo di diffondere il link anche tra i tuoi amici e conoscenti per aiutarci a raccogliere il maggior numero di questionari possibile rispetto agli adolescenti italiani.

Se accetti di partecipare a questo studio, ti chiederemo di compilare un breve questionario online DEL TUTTO ANONIMO creato per valutare l'impatto delle misure restrittive sulla tua vita e sulla tua salute.
Ti serviranno circa 5 minuti per completarlo.
La tua partecipazione allo studio è completamente volontaria e non comporta alcun rischio per la tua salute.
Se ti dovessi sentire a disagio nel rispondere ad una qualsiasi domanda, puoi interrompere l'indagine in qualsiasi momento senza nessun problema.

Il questionario è anonimo e rispetta tutti gli standard etici della ricerca in merito alla protezione dei dati personali che è tutelata sia dalla specifica normativa (DL 101/18), sia dai responsabili del progetto di ricerca.
Il promotore di questo studio è l'Istituto di Ricovero e Cura a Carattere Scientifico, Fondazione Istituto Neurologico Nazionale Casimiro Mondino di Pavia.
Se desideri ricevere informazioni riguardanti la normativa sulla privacy e farci qualche domanda, puoi inviare una mail a questo indirizzo: covid19.ado@mondino.it
Ti risponderemo al più presto.

Grazie per il tuo tempo e per la tua collaborazione!

*Campo obbligatorio

1. Assenso informato *

Acconsento alla raccolta dei Dati Personali per le finalità indicate nell'informativa.

Contrassegna solo un ovale.

- ◯ Acconsento e proseguo la compilazione del questionario
- ◯ Non acconsento e non inizio la compilazione del questionario

Dati personali

2. Inventa un nickname *

____

3. Qual è il tuo anno di nascita? *

Contrassegna solo un ovale.

- ( ) 2003
- ( ) 2004
- ( ) 2005
- ( ) 2006
- ( ) 2007
- ( ) 2008
- ( ) 2009

4. Genere *

Contrassegna solo un ovale.

- ( ) Femmina
- ( ) Maschio

5. Nazione di nascita *

Contrassegna solo un ovale.

- ( ) Italia
- ( ) Estero (Europa)
- ( ) Estero (altro continente)

6. Regione in cui abiti *

Contrassegna solo un ovale.

- Abruzzo
- Basilicata
- Calabria
- Campania
- Emilia Romagna
- Friuli-Venezia Giulia
- Lazio
- Liguria
- Lombardia
- Marche
- Molise
- Piemonte
- Puglia
- Sardegna
- Sicilia
- Toscana
- Trentino-Alto Adige
- Umbria
- Valle d'Aosta
- Veneto

7. Prima dell'emergenza COVID-19 stavi seguendo una terapia psicologica, una psicoterapia e/o visite neuropsichiatriche? *

Seleziona tutte le voci applicabili.

- [ ] No
- [ ] Sedute psicologiche
- [ ] Psicoterapia
- [ ] Visite neuropsichiatriche

8. Durante i mesi di restrizioni hai iniziato o proseguito una terapia psicologica, una psicoterapia e/o visite neuropsichiatriche? *

Contrassegna solo un ovale.

- Sì, ho proseguito le sedute principalmente in presenza
- Sì, ho proseguito le sedute principalmente in modalità online
- Sì, ho iniziato una terapia psicologica/visite neuropsichiatriche in presenza
- Sì, ho iniziato una terapia psicologica/visite neuropsichiatriche in modalità online
- No, durante la pandemia ho interrotto la terapia psicologica/visite neuropsichiatriche
- Non seguivo e non seguo alcun tipo di terapia

9. Nei 6 mesi passati, hai vissuto in prima persona o sei stato/a testimone di eventi traumatici diversi dalla pandemia COVID-19? *

Seleziona tutte le voci applicabili.

- [ ] No
- [ ] Testimone o vittima di violenze domesticheIncidenti
- [ ] d'auto o altri incidenti
- [ ] Incendi
- [ ] Testimone di disastri
- [ ] Testimone o vittima di crimini violenti (rapina o sparatoria)
- [ ] Reazione a notizie traumatiche
- [ ] Traumi di guerra o terrorismo
- [ ] Minaccia di morte
- [ ] Morte reale o minaccia di morte di un membro della tua famiglia/amico stretto
- [ ] Abuso fisico o sessuale
- [ ] Abuso fisico o sessuale subito da un membro della tua famiglia/amico stretto
- [ ] Grave lesione
- [ ] Grave lesione subita da un membro della tua famiglia/amico stretto

10. Hai contratto la malattia da COVID-19? *

Contrassegna solo un ovale.

- No
- Si

11. Se sì, quando hai contratto la malattia?

Contrassegna solo un ovale.

- Novembre 2019
- Dicembre 2019
- Gennaio 2020
- Febbraio 2020
- Marzo 2020
- Aprile 2020
- Maggio 2020
- Giugno 2020
- Luglio 2020
- Agosto 2020
- Settembre 2020
- Ottobre 2020
- Novembre 2020
- Dicembre 2020
- Gennaio 2021
- Febbraio 2021
- Marzo 2021
- Aprile 2021
- Altro: ____________________

12. Attualmente, quanto ti senti stressato/a a causa delle restrizioni? *

Contrassegna solo un ovale.

	0	1	2	3	4	5	6	7	8	9	10	
Per nulla												Moltissimo

13. Se ti senti stressato/a, cosa ti provoca maggiormente stress?

____

14. Al momento ti senti più o meno stressato/a rispetto alla prima ondata di diffusione del COVID-19 (marzo-maggio 2020)? *

Contrassegna solo un ovale.

- Più stressato/a rispetto a prima
- Meno stressato/a rispetto a prima
- Percepisco un livello di stress uguale a quello provato in quel periodo

15. Ti capita spesso di avere pensieri spiacevoli e/o negativi rispetto all'epidemia da COVID-19 a cui non riesci ad evitare di pensare? Mai, occasionalmente o spesso? *

Contrassegna solo un ovale.

	0	1	2	
Mai				Spesso

16. Ti capita spesso di fare sogni spiacevoli o incubi i cui contenuti o le emozioni avevano a che fare con il COVID-19? Mai, occasionalmente o spesso? *

Contrassegna solo un ovale.

	0	1	2	
Mai				Spesso

17. Ti capita di rivivere gli eventi spiacevoli causati dal COVID-19 a distanza di giorni dall'accaduto come se ti stessero succedendo di nuovo in quel momento (per es. flashback)? Mai, occasionalmente o spesso? *

Contrassegna solo un ovale.

	0	1	2	
Mai				Spesso

18. Se succede qualcosa che ti ricorda l'episodio in cui sei stato/a coinvolto/a a causa del COVID-19 provi una intensa sofferenza psicologica? Mai, occasionalmente o spesso? *

Contrassegna solo un ovale.

	0	1	2	
Mai	◯	◯	◯	Spesso

19. Ti senti ancora capace di provare sentimenti positivi (per es. amore, felicità, soddisfazione)? Mai, occasionalmente o spesso? *

Contrassegna solo un ovale.

	0	1	2	
Mai	◯	◯	◯	Spesso

20. Se succede qualcosa che ti ricorda l'episodio in cui sei stato/a coinvolto/a a causa del COVID-19, manifesti reazioni d'allarme esagerate? (es. aumento del battito cardiaco, difficoltà a respirare) *

Contrassegna solo un ovale.

◯ Sì

◯ No

21. Hai mai la sensazione di essere irreale, come se fossi all'esterno del tuo stesso corpo? Di sentirti confuso/a rispetto allo scorrere del tempo? Mai, occasionalmente o spesso? *

Contrassegna solo un ovale.

	0	1	2	
Mai	◯	◯	◯	Spesso

22. Se hai risposto occasionalmente o spesso alla domanda precedente, queste sensazioni erano presenti anche prima della pandemia da COVID-19?

Contrassegna solo un ovale.

- Sì
- No

23. Ti sembra che alcuni ricordi di quanto accaduto a causa del COVID-19 siano svaniti dalla tua mente o ci sono parti o dettagli che non riesci a ricordare? Mai, occasionalmente o spesso? *

Contrassegna solo un ovale.

	0	1	2	
Mai				Spesso

24. Da quando si è diffusa la pandemia da COVID-19, ti capita di pensare di essere cattivo/a, oppure che non ci si possa fidare di nessuno, o che il mondo sia assolutamente pericoloso? *

Contrassegna solo un ovale.

- Sì
- No

25. Ti capita di dare la colpa a te stesso o agli altri per ciò che sta succedendo (pandemia COVID-19)? *

Contrassegna solo un ovale.

- Sì
- No

26. Da quando si è diffusa la pandemia da COVID-19, ti capita di sentirti sempre arrabbiato/a, impaurito/a, in colpa? *

Contrassegna solo un ovale.

- Sì
- No

27. Cerchi di evitare ricordi spiacevoli, pensieri o sentimenti relativi o associati a quello che ti è successo a causa del COVID-19? Mai, occasionalmente o spesso? *

Contrassegna solo un ovale.

	0	1	2	
Mai				Spesso

28. Cerchi di evitare persone, luoghi, conversazioni, attività, oggetti, situazioni che attivano ricordi o pensieri correlati al COVID-19? Mai, occasionalmente o spesso? *

Contrassegna solo un ovale.

	0	1	2	
Mai				Spesso

29. Ti senti più sensibile che in passato rispetto a potenziali minacce? Mai, occasionalmente o spesso? *

Contrassegna solo un ovale.

	0	1	2	
Mai				Spesso

30. Hai difficoltà nel tenere a mente quello che stai facendo? Ti è più difficile fare i compiti o attività che trovi divertenti da quando si è diffuso il COVID-19? Mai, occasionalmente o spesso? *

Contrassegna solo un ovale.

	0	1	2	
Mai	◯	◯	◯	Spesso

31. Senti di essere più nervoso/a? Ad esempio, anche un colpo di tosse o uno starnuto ti spaventa tantissimo? Mai, occasionalmente o spesso? *

Contrassegna solo un ovale.

	0	1	2	
Mai	◯	◯	◯	Spesso

32. Da quando è iniziata la pandemia da COVID-19, ti capita di mettere in atto comportamenti spericolati o autodistruttivi? *

Contrassegna solo un ovale.

◯ Sì

◯ No

33. Ti è capitato, negli ultimi 6 mesi, di avere la sensazione che l'immaginazione ti faccia brutti scherzi, come vedere o sentire cose che gli altri non sembrano percepire e che ti disorientano (es. visioni)? Mai, occasionalmente o spesso? *

Contrassegna solo un ovale.

	0	1	2	
Mai	◯	◯	◯	Spesso

34. Se hai risposto occasionalmente o spesso alla domanda precedente: queste sensazioni erano presenti anche prima della pandemia da COVID-19?

Contrassegna solo un ovale.

- Sì
- No

35. Negli ultimi 6 mesi hai fatto uso di sostanze stupefacenti o alcol in modo abituale (es. 2-3 volte alla settimana)? *

Contrassegna solo un ovale.

- Sì
- No

36. Le difficoltà a cui hai risposto SPESSO alle domande precedenti, da quanto tempo sono presenti?

Contrassegna solo un ovale.

- Da 1 a 3 giorni
- da 4 giorni a 30 giorni
- maggiore di 30 giorni

37. Queste difficoltà stanno influenzando la tua vita?

Contrassegna solo un ovale.

- NO; non ho nessun problema a casa o con gli amici; partecipo ad attività e ho interessi.
- RARAMENTE; sicuro a casa o con gli amici ma raramente mi agito in modo lieve.
- OCCASIONALMENTE; sicuro a casa o con gli amici ma mi agito a seguito di una situazione stressante
- ALCUNI PROBLEMI; chi mi conosce davvero bene può essere preoccupato per me
- QUALCHE PROBLEMA EVIDENTE; i problemi sono evidenti a tutti ma solo in alcune situazioni
- PROBLEMI EVIDENTI; diversi problemi che mi fanno stare male nella maggior parte delle situazioni
- PROBLEMI SERI; sto molto male e vado in crisi in alcune situazioni PROBLEMI
- GRAVI; sto molto male e sono sempre in crisi
- COMPROMISSIONE MOLTO GRAVE; sto molto male e ho bisogno che qualcuno mi aiuti e stia con me
- ESTREMAMENTE COMPROMESSO; sto così male che ho bisogno di una costante supervisione

38. In quest'ultimo periodo hai mai avuto la sensazione di paura improvvisa e incontrollabile associata a sintomi di malessere fisico (attacco di panico)? *

Contrassegna solo un ovale.

- Sì, una sola volta
- Sì, più di una volta
- No

39. Se sì, ti è mai capitato che a causa degli attacchi di panico evitassi di fare qualcosa o avessi paura che gli attacchi si ripresentassero?

Contrassegna solo un ovale.

- Sì
- No

40. Negli ultimi 6 mesi ti è capitato di preoccuparti eccessivamente per tante cose, magari di più rispetto ai tuoi coetanei, senza riuscire a controllare questa preoccupazione? *

Contrassegna solo un ovale.

- Sì
- No

41. Se sì, ti è capitato che ansia e preoccupazione fossero associate a sintomi come tensione, affaticamento, problemi di concentrazione e memoria, irritabilità e alterazioni del sonno?

Contrassegna solo un ovale.

- Sì
- No

42. Nelle ultime 2 settimane hai sentito di essere molto triste, giù di morale, vuoto/a, oppure hai sentito più spesso di aver voglia di piangere? *

Contrassegna solo un ovale.

- Sì, 4 o più giorni a settimana
- Sì, meno di 4 giorni a settimana
- No

43. Nelle ultime 2 settimane hai sentito di essere molto svogliato/a, senza energie e/o particolarmente annoiato/a? Senti che le cose che ti piacevano prima ora non ti piacciono più o ti piacciono meno di prima? *

Contrassegna solo un ovale.

- Sì, 4 o più giorni a settimana
- Sì, meno di 4 giorni a settimana
- No

44. Nelle ultime 2 settimane ti è capitato di aver difficoltà a concentrarti e prestare attenzione alle cose? *

Contrassegna solo un ovale.

- Sì, 4 o più giorni a settimana
- Sì, meno di 4 giorni a settimana
- No

45. Nelle ultime 2 settimane ti sei sentito/a irrequieto/a o, al contrario, rallentato/a? *

Contrassegna solo un ovale.

- Sì, 4 o più giorni a settimana
- Sì, meno di 4 giorni a settimana
- No

46. In quest'ultimo periodo il tuo appetito ti sembra diverso? *

Contrassegna solo un ovale.

- No
- Sì, ho meno appetito ma il mio peso non è cambiato
- Sì, ho meno appetito e il mio peso si è notevolmente ridotto
- Sì, ho più appetito ma il mio peso non è cambiato
- Sì, ho più appetito e il mio peso è notevolmente aumentato

47. In questo periodo com'è il tuo sonno per la maggior parte dei giorni? *

Contrassegna solo un ovale.

- La notte dormo regolarmente e mi sento riposato/a
- Non vado a dormire tardi, ma impiego più di un'ora ad addormentarmi
- Mi sveglio spesso durante la notte e fatico a riaddormentarmi
- Mi sveglio presto al mattino e non mi riaddormento più
- La notte non dormo abbastanza e faccio dei riposini (anche di ore) durante il giorno

48. In quest'ultimo periodo hai mai pensato che sarebbe meglio morire? *

Contrassegna solo un ovale.

- Mai
- Sì, ogni tanto
- Sì, spesso

49. In quest'ultimo periodo ti sei sentito/a talmente male da pensare di farti del male volontariamente? *

Contrassegna solo un ovale.

- No
- Sì, l'ho solo pensato
- Sì, mi sono fatto/a del male ma senza gravi conseguenze mediche
- Sì, mi sono fatto/a del male con gravi conseguenze mediche (es. bisogno di ricovero, pronto soccorso, punti di sutura...)

50. In quest'ultimo anno ti è capitato di avere eccessivi scoppi di rabbia verbali e/o fisici? *

Contrassegna solo un ovale.

- No
- Sì, a volte
- Sì, tutti i giorni

51. Da quando si è diffusa la pandemia da COVID-19 ti capita di sentire meno il desiderio di incontrare i tuoi amici e di provare una sensazione di "distacco" nei loro confronti? *

Contrassegna solo un ovale.

- Sì
- No

52. Se non fossero in vigore le misure restrittive che impediscono di uscire, preferiresti uscire o rimanere comunque a casa? *

Contrassegna solo un ovale.

- Uscire
- Rimanere a casa

53. Prima dell'emergenza COVID-19 cosa preferivi? *

Contrassegna solo un ovale.

- Uscire
- Rimanere a casa

54. Ora che le misure restrittive sono state allentate ti senti... (più di una risposta possibile) *

Seleziona tutte le voci applicabili.

- [ ] Contento/a perché non vedi l'ora di uscire e incontrare amici/altre persone
- [ ] Spaventato/a perché non credi di essere pronto/a ad uscire e incontrare amici/altre persone
- [ ] Timoroso/a per la possibilità di contrarre il COVID-19
- [ ] Preoccupato/a per il ritorno a scuola

55. Secondo te cosa potrebbero fare gli adulti per aiutare i ragazzi della tua età?
